# Relationship Between Performance Characteristics and the Selection Process in Youth Soccer Players

**DOI:** 10.2478/hukin-2014-0021

**Published:** 2014-04-09

**Authors:** Carlos Lago-Peñas, Ezequiel Rey, Luis Casáis, Maite Gómez-López

**Affiliations:** 1 Department of Sports Sciences, Faculty of Education and Sports Sciences, University of Vigo, Pontevedra, Spain.; 2 Faculty of Physical Activity and Sports Sciences, European University of Madrid, Spain.

**Keywords:** soccer, physical fitness, muscle power, aerobic endurance, speed

## Abstract

The purpose of this study was to establish the anthropometric and physical profiles of elite young soccer players according to their playing position, and to determine their relevance for the selection process. One hundred and fifty-six young male soccer players participated in the study. Players were classified into the following groups: Goalkeepers (n=16), Central Defenders (n=26), External Defenders (n=29), Central Midfielders (n=34), External Midfielders (n=28), and Forwards (n=23). Anthropometric variables of participants (body height, body mass, body mass index, 6 skinfolds, 4 diameters, and 3 perimeters) were measured. Participants performed the Yo-Yo test, sprint tests (30 m flat sprint and Balsom agility test) and 2 jump tests (countermovement jump and the Abalakov test). At the end of the season, the technical staff of the club selected some of the players to continue playing on the same team and the rest were not selected. The results show that heavier and taller outfield players performed better in vertical jumps and sprint tests, whereas leaner outfield players performed better in the Yo-Yo test. Fat percentage of selected players was lower than that of the non-selected ones. The rest of the body components were similar in the selected and non-selected players within each playing position. Moreover, the selected players performed slightly better than the non-selected players in the physical test, but these differences were not statistically significant.

## Introduction

Talent identification has long been of great interest to sports coaches and administrators. There are various reasons for this, principal among them being prediction of success in adult elite competition and determination of appropriate development processes to achieve adult success (Reilly et al., 2000). Besides technical and tactical skills, which are of primary importance in soccer, anthropometrical and physical characteristics are actually crucial to discriminate talented from non-talented soccer players.

Anthropometric measures and physical performance tests are regularly performed in soccer academies, both for aiding selection/detection (Reilly et al., 2000) and training monitoring purposes (Buchheit et al., 2012). Many studies have been published reporting these characteristics of professional soccer players of different positions (Barros et al., 2007; Bloomfield et al., 2007; Bradley et al., 2009; Dellal et al., 2011; Di Salvo et al., 2007; Rampinini et al., 2007; Wong et al., 2008). However, similar studies investigating the positional differences in physical performance among youth soccer players are limited, and the results have been inconsistent (Gil et al., 2007; Lago-Peñas et al., 2011; Le Gall et al., 2010; Malina et al., 2004; Silva et al., 2013; Alemdaroğlu, 2012). Malina et al. (2004) studied elite youth soccer players aged 14 years with 4.5 years of training experience and found that there were no differences between Defenders, Midfielders, and Forwards in vertical jumps, 30-m sprint time, and intermittent aerobic endurance. Another study by Gil et al. (2007) reported that Goalkeepers had a significantly lower aerobic capacity than did Defenders, Midfielders, and Forwards. In addition, Forwards had the best performance in the 30-m sprint and vertical jumps compared with Goalkeepers, Defenders, and Midfielders. Wong et al. (2008) studied seventy U14 male soccer players with 5 years’ of training experience and found that there were significant positional differences in anthropometry among youth soccer players, but no significant positional differences in physiological and fitness performances (maximal vertical jump, ball shooting, 30-m sprint, and VO_2_max). Reilly et al. (2000) studied thirty-one youth male soccer players and showed that elite players were significantly leaner, possessed more aerobic power and were more tolerant to fatigue than sub-elite soccer players.

Moreover, the majority of these studies have classified the anthropometric and physical profiles of young soccer players according to 4 playing roles: forwards, midfielders, defenders, and goalkeepers. It is possible that important information regarding positional difference in youth players might be masked. In fact, the physical profile of the contemporary elite players has been described according to six positional roles: Goalkeepers (GK), Central Defenders (CD), External Defenders (ED), Central Midfielders (CM), External Midfielders (EM), and Forwards (F). It has been reported that in a professional match, a CM covers a significantly greater distance than does a CD or a F; whereas a F performs significantly more sprints than a CD or a CM does (Barros et al., 2007; Bradley et al., 2009; Di Salvo et al., 2008; Rampinini et al., 2007; Rampinini et al., 2009).

Consequently, the first aim of the present study was to establish the anthropometric and physical profiles of youth soccer players according to their playing positions. The second aim was to determine whether certain physical and anthropometric characteristics discriminated between the selected and non-selected players. These findings could facilitate talent identification, the selection of youth players, and a training design.

The hypothesis is that characteristic anthropometric differences exist between different playing positions. On the other hand, we believe that there are no differences in physical performance between different playing positions because in comparison with high-level adult soccer, in youth soccer, match intensity and duration are of a lower level, weekly training volume and intensity are lower, and youth players accumulate fewer years of training compared to adults.

## Material and Methods

### Participants

One hundred and fifty-six youth male soccer players participated in the study, which was conducted near the end of the first half of the soccer season (weeks 19–20 of the 42-week season). They were members of regional representative teams competing at the highest level of competition for their category in Spain. The number of players in each team and their average age are shown in Table 1. Players were classified according to their playing roles into 6 groups: GK (n=16), CD (n=26), ED (n=29), CM (n=34), EM (n=28), F (n=23), based on the different activity on the pitch, and the primary area in which this activity was carried out (Di Salvo et al., 2007).

### Procedures

The training season starts in August with 8 weeks of physical conditioning, including endurance training in particular. This is followed by soccer-specific training. Players trained for 90 minutes 3 times per week and played a match during the weekend. Each soccer training session generally consisted of a 15-minute warm up, 20 minutes of technical training, 20 minutes of tactical training, 30 min simulated competition, and a 5 min cool-down. Within the team, players of all the different positions trained together except for the GKs who dedicated the technical training session to specific training. The study was conducted according to the Declaration of Helsinki, and the protocol was fully approved by the Clinical Research Ethics Committee. All players and their parents were properly informed of the nature of the study without being informed of its detailed aims. Each player and his parents or guardians were informed of the experimental risks, and both signed an informed consent form before the investigation.

### Measures

#### Anthropometry, Somatotype, and Body Composition

Body height (cm) and body mass (kg) of each player were measured and the body mass index (BMI) was calculated (kg·m^−2^). Skinfolds (mm) were measured at 6 sites: triceps, subscapular, abdominal, suprailial, thigh, and lower leg, using a skinfold calliper (Harpenden, UK). Each individual measurement and the sum of the 6 measurements were used for analysis. The circumferences of the upper arm, thigh, and lower leg were measured (cm), as well as the following 4 diameters (cm): biepicondylar humerus (elbow), biestyloid at the wrist, biepicondylar femur (knee), and bimaleolar in the ankle. All the measurements were made following the guidelines outlined by the International Society for the Advancement of Kinanthropometry (ISAK). Afterwards, body mass, percentages of fat, bone, and muscle were calculated in order to evaluate body composition, using the formulas of Faulkner (1968), Rocha (1975), Wurch (1974) and Matiegka (1921). The endomorphy, mesomorphy, and ectomorphy components of the somatotype were also calculated.

### Physical Tests

#### Yo-Yo test

The Yo-Yo Intermittent Endurance Test was designed to evaluate the ability to perform intense exercise repeatedly during prolonged intermittent exercise (Bangsbo and Michalsik, 2002). In the test each participant performed a series of 20-m shuttle runs at a pace set by an audio metronome from a calibrated CD player (Sony CFD-V7), with a standard rest interval between shuttles (5 s). The time allowed for the shuttles was progressively decreased, while the speed was increased. The test was terminated when the subjects failed twice to reach the starting line or the participant felt unable to complete another shuttle at the dictated speed.

#### Sprint Time

**Figure 1.**
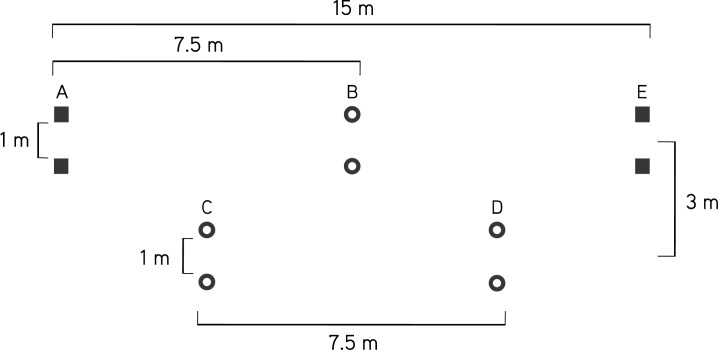
Balsom agility test course. Players start at point A, and sprint to the cones at point B. They turn at point B, sprint back through point A, turn to the left and sprint through point C to point D. They turn at point D and then sprint back through C, turn to the right and sprint through point B to the finishing gate shown at point E. All distances are indicated on the diagram.

The soccer players performed 2 tests on a running track: a 30-m flat sprint to estimate velocity and the Balsom’s test (Balsom, 1994) to estimate agility (Figure 1). The players were asked to complete a 10-minute specific warm-up including several accelerations to decide which foot they would have to set on the starting line for the sprint start. The players had to start from a standing position placing their forward foot just behind the starting line and their rear foot on the pedal. Sprint times were measured with an infrared photo-electronic cell (Speedtrap II Wireless Timing System; Brower Timing Systems, Draper, UT). There were 2 trials in each test, and 3-minute recovery was allowed between each trial. The fastest 30 m sprint and agility times were selected for analysis.

#### Jump Tests

To measure explosive strength of the lower extremities, players performed 2 jump tests (countermovement jump [CMJ], and the Abalakov test [ABA] using a jump mat (Ergojump, Bosco-Systems, Italy)). The CMJ was performed standing with straight legs and performing a jump beginning with a counter movement down to a knee angle of 90 degrees. The hands were held on the hips during the jump to avoid any effect on arm-swing. The Abalakov jump assesses explosive strength, plus the use of elastic energy, as well as the coordinate capacity using trunk and upper limbs. Jump height was determined based on flight time. Each player performed 2 jumps interspersed with a 1-minute rest between each jump. The height of the jumps was measured in cm, and the best jump of each modality was selected.

### Selection Process of the Soccer Players

At the end of the season, three experts of the technical staff of the club selected some of the players to continue playing on the same team and the rest were not selected according to their performance during the competitive matches of the season as well as the technical and tactical performance obtained on the F-MARC test battery (Rösch et al., 2000). The technical staff were given a list of players and asked to assess their performance according to one of two levels: (1) selected players performed as expected or above their normal standard and (2) non-selected players performed below their normal standard. In this study, the anthropometric and physical characteristics of the selected and the non-selected players were analyzed in order to identify the variables associated with selection for a given position on the field.

### Analysis

The results were analyzed using the SPSS software (version 20.0; SPSS, Inc., Chicago, IL). A 2-way analysis of variance (ANOVA) was used to evaluate group differences. Post hoc comparisons were determined by the Scheffé test when the variances were equal and by the Games-Howell test when they were not equal. To analyze differences between the selected and non-selected players within each playing position, the Student’s t-test was performed using each variable. An alpha of p ≤ 0.05 was used for statistical significance. The results are presented as mean and *SD*.

The reliability of each test was assessed by intraclass correlations (ICCs) and coefficients of variance (CV). The results show that these tests were highly repeatable: CMJ (ICC = 0.93; CV=4.5%; n = 161), ABA (ICC = 0.96; CV=3.5%; n = 161), 30-m sprint (ICC = 0.95; CV = 2.3 %; n = 161), 30-m sprint with 10 cones (ICC = 0.94; CV = 2.6 %; n = 161). Although the repeatability of the Yo-Yo test cannot be calculated from the present study as it was performed only once, previous studies have shown that there was no significant differences between test-retest distance coverage.

## Results

The average values of body mass and body height are shown in Table 2. CDs were the tallest players and they were also the heaviest compared to EDs, EMs, and CMs (p<0.05).

The BMI (kg·m^−2^) of the CDs was higher compared to that of the EDs, EMs, and CMs (p<0.05).

The endomorphy values were higher in the CDs and GKs compared to EMs (p<0.05). In addition, EDs and Fs presented the highest ectomorphy and mesomorphy values, respectively.

**Figure 2.**
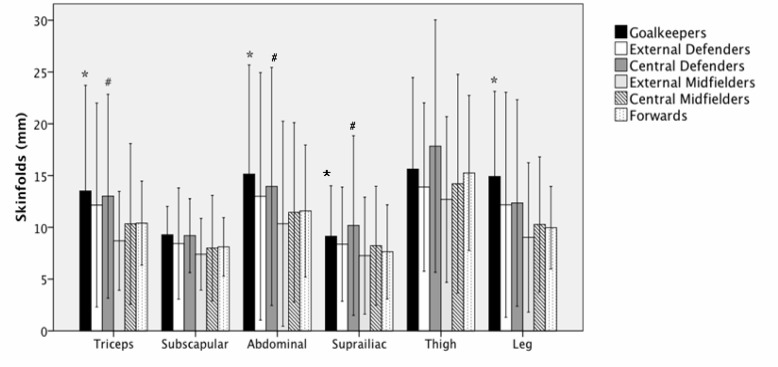
Measurements of different skinfolds of soccer players, classified according to their playing positions (mean ± SD). ^*^GKs vs. EM, p<0.05. ^#^ EM vs. CD, p<0.05.

EMs had less fat than did GKs in the triceps, abdominal, suprailiac, and leg sites (p<0.05). Also, the group of EMs had less fat than did CDs in the triceps, abdominal, and suprailiac skinfolds (p<0.05). When all the skinfolds were added, EMs (55.43 ± 16.37 mm) were found to be leaner than CDs (78.38 ± 28.37), and GKs (89.67 ± 33.57) (p<0.05). In addition, GKs were found to be heavier than CMs (63.12 ± 19.79 mm), and Fs (62.94 ± 10.61 mm) (p<0.05) (Figure 2).

The fat, muscle, and bone mass of the CDs were higher compared to that of the EDs, EMs, CMs, and Fs (p<0.05). Also the fat, and muscle mass of GKs were higher compared to EDs, EMs, CMs, and Fs (p<0.05), and EDs, CMs, and EMs (p<0.05), respectively. Fat percentage was also higher in the CDs compared to the EMs, and Fs (p<0.05) and in the GKs compared to the EMs (p<0.05) (Table 3).

**Figure 3.**
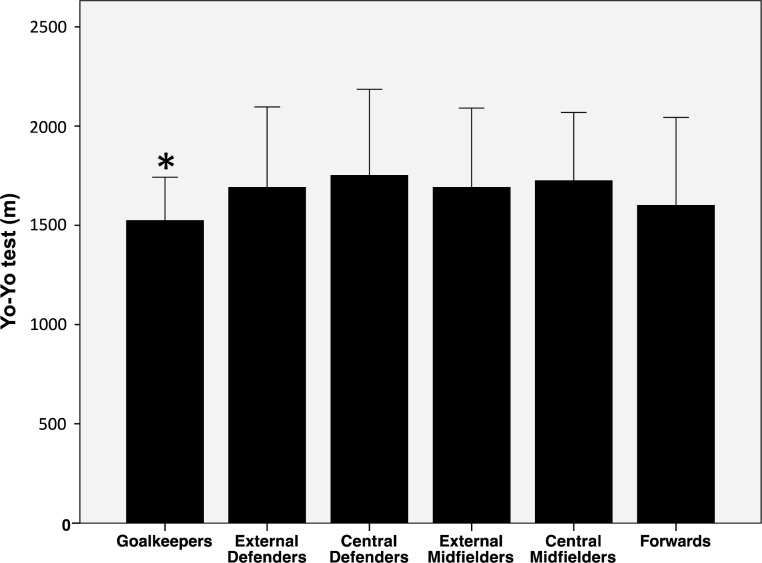
Yo-Yo test performance by soccer players classified according to their playing position (mean ± SD). ^*^GK vs. ED, CD, EM, and CM, p<0.05.

GKs had the lowest performance compared to the rest of the groups in the Yo-Yo test (p<0.05) (Figure 3).

In the Balsom agility test, CDs were faster than the other groups.

In the 2 jump tests, CDs showed the best performance of all the positional groups, but these differences were not statistically significant. EDs and CMs produced the shortest jumps in the CMJ and Abalakov test, respectively.

Fat percentage of selected players was lower than that of the non-selected players. These differences were significant only for the CDs (p<0.05). The rest of the body components were similar in the selected and non-selected players within each playing position.

**Figure 4.**
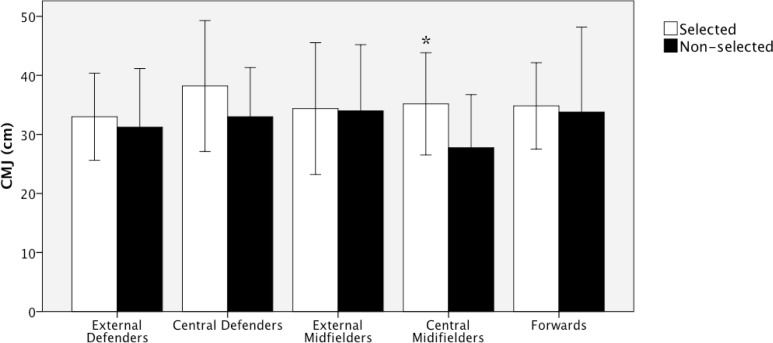
Differences in the counter movement jump test (CMJ) between the selected and non-selected players for each playing position (mean ± sd). ^*^Selected vs. non-selected, p<0.05.

The selected players reached better results in the 2 jump tests than their counterparts; but these differences were statistically significant only for the CM test (p<0.05) (Figure 4).

## Discussion

The aim of this study was to establish the anthropometric and physical profiles of elite youth soccer players according to their playing position, and to determine their relevance for the selection process. The major finding of this study is that anthropometric characteristics of youth soccer players differed according to the playing positions, especially for EMs (the leanest and shortest) and for the GKs and CDs (the tallest, heaviest, and with largest skinfolds). However, there were no differences in physical performances. Finally, it appeared that successful players were leaner and more muscular than unsuccessful players.

The results support our hypothesis that there were significant positional differences in anthropometry such as body mass, body height, and BMI. Specifically, GKs (64,3 kg, 1.70 m) and CDs (68,2 kg, 1.73 m) were the heaviest and tallest players, and EDs (55.8 kg, 1.64 m), EMs (54,5 kg, 1.64 m), and Fs (61.5 kg, 1.67 m) were the lightest and shortest. These results partially agree with those of previous studies on U11 to U17 soccer players, which showed that Fs were lighter than CDs, EDs and GKs, but heavier than CMs and EMs, whereas Fs were shorter than CMs, EMs, GKs, CDs and EDs (Carling et al., 2009; Da Silva et al., 2008; Gil et al., 2007; Gravina et al., 2008; Lago-Peñas et al., 2011; Malina et al., 2000; Wong et al., 2009). In terms of BMI, the present results differed from the previous study (Wong et al., 2008) that reported that GKs had higher values than CDs, EDs, Fs, CMs and EMs among professional players. While talent selection is based on many aspects of performance, the present results suggest that certain antrophometric assessment data are important in determining whether already highly selected elite youth soccer players are successful or not in achieving higher standards of play, such as taller and heavier players are more suitable to be a GK and CD and shorter and lighter players are more suitable to be an ED and EM (Stølen et al., 2005). The results of the present study support the fact that U20 players with different playing positions are characterized by different anthropometry such as body mass, body height, and BMI.

The results of the present study generally support our hypothesis that there are no positional differences in physical variables. Particularly, no significant differences were found in physical variables except for the Yo-Yo test where GKs had the lowest performance compared to the rest of the groups (p<0.05). Similar results have been published in other studies (Gil et al., 2007; Wong et al., 2009).

This study agreed with previous ones, which found no statistical difference in jump height among GKs, CDs, EDs, CMs, EMs and Fs of U13 to U20 soccer players (Gil et al., 2007; Malina et al., 2000; Wong et al., 2009), although CDs showed the best performance of all positional groups. However, Stolen et al. (2005) reported that at the professional level, GKs had the highest jump height and CMs and EMs had the lowest compared with CDs, EDs, and Fs.

Fs and CDs were the fastest players in the 30-m sprint and the Balsom agility tests. However, these differences were non-significant in the 30-m sprint test. This disagrees with the performance characteristics of elite adult soccer players. Therefore, it is reasonable to state that at the U15 to U20 level, sprint performance is not significantly different among playing positions, but when it is approaching the professional level, positional differences exist, where Fs become the fastest players as they cover the greatest distance at high speed during games.

Other authors have observed similar results (Gil et al., 2007; Wong et al., 2009). GKs should also be fast and agile, buy they did not perform that well in the sprint tests. Perhaps these 2 tests are not the most appropriate for measuring their fitness. In fact, GK movements are much shorter and their sprinting distance has been reported to be between only 1−12 meters long (Di Salvo et al., 2008). Thus, more specific tests should be designed to measure the capacities and abilities of GKs.

In general, the results show that heavier and taller youth outfield players performed better in vertical jumps and sprint tests, whereas leaner players performed better in the Yo-Yo test. In agreement with this, Malina et al. (2005) found that body mass was the most significant predictor in 30-m sprint performance and body height was the significant predictor of vertical jump performance. Moreover, players in the present study with higher BMI values performed better in vertical jumps and 30-m sprints but had poor performances in the Yo-Yo test. Indeed, as suggested by Wong et al. (2009), a high BMI at an equivalent body fat content and height means a higher lean body mass and thus higher muscular mass. This is in favour of strength and power activities but represents a limiting factor for weight-bearing activities such as running endurance efforts. Nevertheless, the present study does not justify such practice of soccer coaches in the long-term process of player development. Indeed, the long-term effect of selecting players based on their anthropometry advantage leads to a strong bias for those players who mature early (heavier and taller) to be selected into professional, semiprofessional, and U15 to U18 national teams (Helsen et al., 2005; Vaeyens et al., 2005), and eventually, the proportion of youth players who are lighter and shorter decreased with increased age from U11 to U16 (Malina et al., 2004; Wong et al., 2009).

The results of the present study show that the selected players are leaner and more muscular than their non-selected counterparts, however these differences were not statistically significant. The rest of the body components were similar in the selected and non-selected players within each playing position. These results agree with the findings of Gil et al. (2007). Furthermore, the selected players performed slightly better than the non-selected players on the physical test, but these differences were not statistically significant. These results are similar to those provided by Gil et al. (2007) and Reilly et al. (2000). Therefore, as suggested by Reilly et al. (2000), other than absolute anthropometry advantages, psychological and soccer-specific skills should also be considered in the selection of youth soccer players for developing future high-class players.

The results suggest that there were significant positional differences in anthropometry such as body mass, height, and BMI. Specially, Gs and CDs were the heaviest and tallest players, and EDs, EMs, and Fs were the lightest and shortest. However, there were no positional differences in physical performances.

This study provides a scientific rationale of the coaches’ practice in selecting youth soccer players according to their anthropometry for short-term benefits and does not justify such practice in the long-term process of player development. In fact, it seems that coaches could receive short-term benefits by employing heavier and taller players for positions (e.g., CD and F) that require higher jumping and sprinting abilities; and players with lower BMI for positions (e.g., ED and EM) that require higher aerobic endurance. Consequently, technical staff should take the present results into account and should not discriminate against younger or late-maturating players who may develop their abilities later. In this context, opportunities need to be provided for smaller and/or later maturing talented boys during adolescence.

Moreover, it appeared that selected players were leaner and more muscular than those not selected. These results suggest that specific training programs in strength, aerobic activities, and speed could be used to improve fitness abilities according to the different physical, anthropometric, and somatotype profiles in youth soccer players. Therefore, training programs need to be modified for youth soccer players, and direct application of the program used by senior players may not be appropriate.

Finally, we acknowledge two major limitations of this study. First, the broad age range of players analyzed (ranged from 15 to 20 years) that makes general conclusions difficult. Second, the number of tests conducted was limited because the test session was completed late in the season when there were high demands on training and competitive time. Coordination, repeated-sprint ability, dribbling and anticipation skills are other discriminating factors between youth players of different standards and positions.

## Figures and Tables

**Table 1:** Age (mean ± SD) and number of players classified according to their playing positions

Team	Age	Players (n)	Goalkeepers	External Defenders	Central Defenders	Extenal Midfielders	Central Midfielders	Forwards
U15	12.97 + 0.49	57	5	10	8	11	13	10
U17	14.92 + 0.56	55	6	12	9	10	10	8
U20	17.57 + 0.96	44	5	7	9	7	11	5
Total	14.97 + 1.97	156	16	29	26	28	34	23
	Age (y)		14.78 ± 2.28	14.44 ± 1.38	15.69 ± 2.25	15.13 ± 1.68	14.87± 2.10	15.16 ± 2.16

**Table 2:** Physical characteristics and somatotype of soccer players (mean ± SD)

	Goalkeepers	External Defenders	Central Defenders	External Midfielders	Central Midfielders	Forwards
Body mass (kg)	64.31 ± 10.17	55.84 ± 10.89	68.22 ± 10.91^*^	54.47 ± 10.91	54.35 ± 12.35	61.50 ± 12.06
Height (cm)	169.89 ± 12.06	164.17 ± 9.76	173.27 ± 10.40^*^	164.13 ± 9.99	161.93 ± 10.76	166.63 ± 10.30
BMI (kg·m^−2^)	21.45 ± 1.32	20.56 ± 2.57	22.56 ± 1.60^*^	19.99 ± 2.09	20.41 ± 2.61	21.93 ± 2.30
Endomorphy	3.5 1 ± 1.41^$^	3.03 ± 1.16	3.23 ± 1.19^†^	2.40 ± 0.75	2.80 ± 0.93	2.70 ± 0.54
Mesomorphy	4.74 ± 1.17	4.94 ± 1.18	4.91 ± 1.27	4.48 ± 0.76	4.91 ± 1.21	5.21 ± 1.09
Ectomorphy	2.79 ± 0.99	2.97 ± 1.08	2.68 ± 0.89	2.88 ± 1.01	2.74 ± 0.97	2.91 ± 0.94

BMI: body mass index

* CD vs. ED, EM and CM, p<0.05.

† CD vs. EM, p<0.05.

$ GK vs. EM, p<0.05.

**Table 3:** Body composition (body mass and percentage, mean ± SD) of soccer players playing in different positions. The residual percentage is always 24.12%, and therefore it has been deleted from the table.

		Goalkeepers	External Defenders	Central Defenders	Central Midfielders	External Midfielders	Forwards
Body mass (kg)	Fat mass	14.11 ± 3.41^†^	11.27 ± 3.24	12.27 ± 3.19^*^	10.47 ± 2.86	10.15 ± 2.74	10.82 ± 1.86
	Muscle mass	20.58 ± 7.92^$^	20.33 ± 6.64	26.62 ± 8.54^*^	19.91 ± 7.30	20.07 ± 6.03	23.95 ± 8.27
	Bone mass	11.88 ± 2.31	11.34 ± 1.80	12.88 ± 2.28^*^	10.86 ± 1.72	11.11 ± 1.86	12.05 ± 1.77
	Residual	14.77 ± 2.43	13.44 ± 2.81	16.43 ± 2.62	13.09 ± 2.98	13.12 ± 2.62	14.64 ± 2.91
Percentage (%)	Fat cont	13.75 ± 3.36^+^	12.28 ± 2.28	13.14 ± 2.81^#^	11.59 ± 1.81	10.94 ± 1.63	11.55 ± 0.95
	Muscle cont	42.84 ± 3.41	43.29 ± 2.21	43.68 ± 3.89	43.91 ± 2.03	44.36 ± 1.49	44.50 ± 1.86
	Bone cont	19.33 ± 1.67	20.33 ± 2.01	19.06 ± 2.88	20.38 ± 2.12	20.59 ± 1.54	19.84 ± 1.86

† GK vs. ED, CM, EM, and F, p<0.05.

$ GK vs. ED, CM, and EM, p<0.05.

+ GK vs. EM, p<0.05.

* CD vs. ED, CM, EM, and F, p<0.05.

# CD, EM., and F, p<0.05.
